# Preparation of Molecular Imprinted Polymer Based on Chitosan as the Selective Sorbent for Solid-Phase Microextraction of Phenobarbital

**DOI:** 10.1155/2022/9027920

**Published:** 2022-07-13

**Authors:** Marzieh Rahimi, Soleiman Bahar, S. Mojtaba Amininasab

**Affiliations:** Department of Chemistry, Faculty of Science, University of Kurdistan, P.O. Box 416, Sanandaj, Iran

## Abstract

This study reports the construction of a novel SPME fiber based on chitosan and glutaraldehyde as coating material composites combined with high-performance liquid chromatography with an ultraviolet detector (HPLC-UV) for extraction and detection of phenobarbital. In this technique, the chitosan biopolymer, as a new coating of SPME fiber, was produced on the stainless-steel wire, using glutaraldehyde and phenobarbital as cross-linker and template, respectively. For comparison, a nonimprinted polymer was created using the same procedure to evaluate fiber selectivity (but without the addition of phenobarbital). The SPME-MIP fiber coating was characterized by field emission scanning electron microscopy, Fourier-transform infrared spectroscopy, and thermal gravimetric analysis. The efficiency of fiber was then improved by adjusting the impact of numerous factors such as pH, extraction time, desorption time, desorption solvent, and stirring rate. The results showed that the proposed fiber has a linear range of 0.01–4 *μ*g·mL^−1^, and detection limit of 7.5 ng·mL^−1^. The average recoveries in the four concentration levels for the spiked river and well water samples were 95.7 and 95.3%, with relative standard deviations of 3.8 and 5.9% for single fiber and between fibers, respectively.

## 1. Introduction

Poisoning from excessive use of any pharmaceutical or chemical (also known as drug poisoning) has become a worldwide problem in recent years [1]. Phenobarbital, a barbiturate derivative, is one of the most commonly prescribed epilepsy medications. Barbiturates have long been utilized as hypnotics, anticonvulsants, anesthetics, and sedatives due to their interaction with the gamma-aminobutyric acid receptor [2]. Barbiturates have narrow therapeutic dosages and severe side effects; therefore, choosing the right antidepressant for each patient is critical to avoid drug poisoning [3]. Barbiturates, on the other hand, are a serious environmental hazard due to their toxicity and long-term persistence in environmental matrices [4]. It was proven that constant exposure to drugs or their metabolites by humans and other living organisms may have a deleterious impact on their health [5]. Therefore, it is critical to develop a sensitive method for analyzing pharmaceutical residues in the blood and/or aquatic environments.

Until today, numerous methods have been used to quantify phenobarbital and barbiturates in medicinal research and biological samples, including liquid chromatography equipped to photodiode-array detection system (LC-PDA) [6], liquid chromatography-mass spectrometry (LC-MS) [7], gas chromatography-mass spectrometry (GC-MS) [8], and capillary electrophoresis (CE). Almost all the identification and quantification methods need to some sample preparation technique (s) before analysis. The sample cleansing techniques improved separation and detection accuracy and precision, while cutting down the assay time and expense. Solid-phase microextraction (SPME) is an adsorptive-based extraction sample preparation process that integrates sampling, isolation, and enrichment into a single step. Due to simplicity and effectiveness, SPME is a popular method in the measurement of diverse analytes from complicated matrices [9]. This method is based on the partitioning of the analyte between the sample and the extracting phase (coated on SPME fiber) [10]. To ensure the procedure's selectivity and sorption capacity, the fiber coating should be chosen carefully. Nowadays, several novel coating materials were developed to boost the efficiency of the extraction procedure because of several constraints of common commercial fibers, such as poor selectivity and stability [11]. Selectivity, durability, porosity, and high surface area are important factors that should be considered while preparing a coating.

The imprinted polymerization method is one of the most useful strategies for designing SPME fibers, because of selectivity, and chemical and thermal stability of final polymers. Polymerization of functional monomer and cross-linking agent in the presence of template molecule creates three-dimensional cavities in the polymer network that match the template [12]. In molecularly imprinted polymers (MIPs), cavities that perfectly match the template molecule build high degree affinity and selectivity toward the target analyte, even in complicated matrices. Classic molecular imprinting approaches suffer from a lot of limitations such as poor binding capacity, limited accessibility to binding sites, target molecule embedding challenges, and problems in eluting the target. To overcome these drawbacks, the development of alternative imprinted polymer production technologies is inevitable [13]. This idea led to the utilization of high-surface-area materials such as graphene [14], magnetic Fe_3_O_4_ [15], and carbon nanotubes [16]. Natural biopolymers have gained increased attention in recent years as a substitute for synthetic materials [17]. This enthusiasm arises from unique features of biopolymers such as ease of processing, nontoxicity, environmental friendliness, high sorption capacity, availability, and low cost, for example, a novel natural nontoxic polydopamine/dialdehyde starch/chitosan (PD/DAS/CHI) coating developed by Cheng et al. for in-tube solid-phase microextraction. This coating demonstrated high sensitivity, stability, and rapidity when utilized to screen hexanal and 2-butanone biomarkers in patients suspected to liver cancer [18]. Alizadeh et al. produced a chitosan-zinc oxide nanorod composite as a novel SPME fiber coating on the surface of fused silica. This technique not only allows for mild synthesis, but it also reduces/eliminates the use of toxic organic solvents [19].

Chitosan, the second most abundant nonsynthetic biopolymer, is created by living organisms such as crustaceans (shells) and fungal (biomass) [20]. Chitosan is nontoxic, biocompatible, and biodegradable polymer and also possesses antimicrobial characteristics. The main studies on chitosan have focused on its use in wastewater treatment [21]. On the other hand, chitosan has been widely used as a functional monomer or a supporting matrix in numerous synthesis research studies, but no study has been published that describes its use as an MIP monomer in SPME [22]. Chitosan has a lot of amino and hydroxyl groups that can interact with the functional groups of template molecule. However, various modifications are required to overcome chitosan's inherent weakness (low mechanical strength) and increase its chemical and physical properties [20]. For example, the utilization of cross-linking compounds (like glutaraldehyde (GLA)) improves the reactivity and stability of chitosan and simultaneously increases the number of active sites within the polymer network GLA, which creates a robust and homogenous network by covalently bonding to the amine groups of the chitosan hydrogel [19]. Until now, phenobarbital and other barbiturates have been determined using a variety of extraction methods [23–26]. For instance, a surface-modified uniform-sized molecularly imprinted polymer has been developed through a two-step swelling polymerization method using polystyrene beads, phenobarbital, 4-vinylpyridine, and ethylene glycol dimethacrylate, as seeds, template, functional monomer, and cross-linker, respectively. The unique HPLC column manufactured by this polymer showed good performance in the analysis of biological samples [25]. Another study employed a multistep swelling method to make uniformly sized magnetic MIP particles. The prepared MIP exhibited a high affinity and binding capacity for the extraction of PB from human serum samples [27]. Synthesis of new magnetic molecular imprinted materials [27], preparation of novel MIPs by sol-gel technology [5, 28], and fabrication of brand-new coatings by green nanoparticles all belong to this concept [29]. In this study, we propose a novel, simple, and effective solid-phase microextraction technique for the determination of phenobarbital in water samples by HPLC-UV through covering of stainless-steel wires (supporting material) by homogenous chitosan biopolymer.

## 2. Experimental

### 2.1. Materials and Methods

Chitosan (purity ≥75–85%), glutaraldehyde (50 wt. % in H_2_O), phenobarbital (purity ≥99.0%), paracetamol (purity ≥99.0%), and caffeine (purity ≥99.0%) were provided by Sigma-Aldrich (Missouri, United States). Acetic acid, methanol, sulfuric acid, hydrochloric acid, sodium hydroxide, sodium hydrogen phosphate, acetonitrile, chloroform, dichloromethane, and ethanol were analytical grades and obtained from Merck (Darmstadt, Germany). HPLC-grade methanol was bought from Merck (Darmstadt, Germany). The stock solution of phenobarbital was prepared in methanol (1000 *μ*g·mL^−1^) and was kept in a refrigerator at 4°C until use. To produce working solutions, stock solution was diluted with double distilled water to make the required concentration.

### 2.2. Apparatus

Separations were carried out by HPLC-UV instrument, consisted of Knauer HPLC system (Berlin, Germany) equipped with a Knauer S1050 pump, a Smartline UV detector 2600, and ClarityChrom Software. Isocratic separation was carried out by a mixture of methanol and water (40 : 60, v/v) as a mobile phase, which was delivered at a flow rate of 0.8 mL·min^−1^ to a Eurospher C_18_ column (250 mm × 4.6 mm i.d., 5 *μ*m). The UV detector was set at 230 nm. A consort C1010, Cleaver Scientific (Warwickshire, UK), pH meter was employed for pH measurements. Fourier-transform infrared spectroscopy analysis was performed by a Thermo Nicolet 6700 (Massachusetts, United States). Scanning electron microscopy (SEM) images were taken using the Mighty-8 instrument (TSCAN, Prague) field emission scanning electron microscope. Scanning electron microscopy-energy-dispersive spectroscopy (SEM-EDX) analyses were carried out using a Mighty-8 instrument (TSCAN, Prague). Thermogravimetric analysis (TGA) was performed using a DuPont instrument (TGA 951) between room temperature and 700°C with a heating rate of 10°C·min^−1^ in a nitrogen atmosphere. The stirring was performed by a Pole IDEAL PARS Co. magnetic stirrer (Iran).

### 2.3. Surface Activation of Stainless-Steel Wire

Stainless-steel (SS) wires were chosen as supporting materials because they improve the physical stability and lifespan of SPME fibers when compared to commercially available fibers. In order to surface activation of (SS) wires, the wires (300 *μ*m O.D., and 5.0 cm-long) (Vita, Needle Co., Needham, Ma, USA) initially were washed in an acetone/methanol ultrasonic bath. After 10 minutes, the wires were rinsed with double distilled water (DDW) to eliminate the organic chemical contaminants. The inert surface of the wires was then activated by oxidizing through dipping it in a sulfuric acid solution (2 M). After 4 hours, DDW was used to rinse the product [30].

### 2.4. Preparation of the MIP and NIP Cross-Linked Chitosan/Glutaraldehyde

**Scheme 1:**
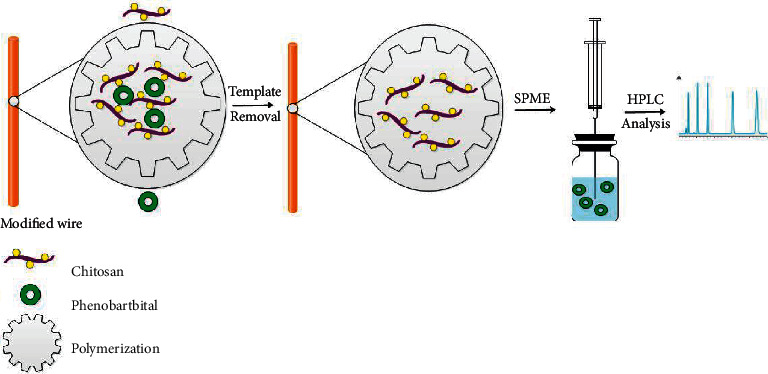
Schematic image of the SPME-MIP procedure.

The molecularly imprinted chitosan/glutaraldehyde fiber was produced as follows: At 60°C, 0.1 g of chitosan was dissolved in 11 mL of 2% acetic acid aqueous solution. Then, 0.05 g of phenobarbital was dissolved in 1 mL ethanol and added to the aforementioned solution. After 1 hour of stirring, 300 *μ*L of glutaraldehyde solution (10%) was added to the mixture [31]. Following that, the modified SS wires (which had previously been installed in parallel on a silicon rubber) were immersed in the mixture and stirred at room temperature. After 3 hours, the MIP-coated fibers were pulled out from solution and dried at 60°C. To increase the thickness of coating, the fibers were immersed in fresh polymerization solutions, and the process was repeated several times. Following further studies showed that four times polymerization yielded the best outcomes. To remove the template from fibers, the SPME-MIP fibers were soaked in methanol for 30 minutes, and the process was repeated several times until no phenobarbital was detected in the soaking elution by HPLC-UV. The nonimprinted polymer (NIP) SPME fibers were prepared by the same procedure but without the addition of the template. The SPME-MIP synthesis technique is illustrated schematically in Scheme 1.

### 2.5. Extraction Procedure

The SPME-MIP fiber was directly immersed in a 10-mL glass vial containing 7 mL phenobarbital standard aqueous solution by using a handmade SPME device to extract the analyte. The fiber was taken out and rinsed with DDW to remove the unbounded matrix components after 20 minutes of agitation in room temperature (600 rpm). The MIP coatings were eluted with 2 mL of methanol (as a desorption solvent) for 15 min at room temperature to desorb the analyte. Then, the solvent was evaporated until a stream of nitrogen and reconstituted in 100 *μ*L of methanol. Finally, 20 *μ*L of the elution solvent were loaded into the HPLC for analysis.

## 3. Results and Discussions

### 3.1. Characterization of the MIP Cross-Linked Chitosan/Glutaraldehyde Fibers

**Figure 1:**
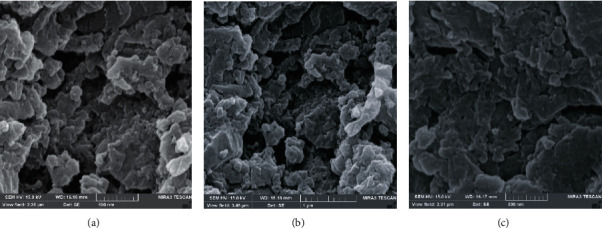
SEM image of chitosan SPME-MIP fiber with the magnification of 60.0kx (a), 90.0kx (b), and SEM image of NIP (c).

The morphology of SPME-MIP fiber coating using chitosan as the functional monomer and the corresponding SPME-NIP fiber coatings were examined via SEM, FTIR, TGA, and EDX. The SEM images showed that the SPME-MIP coatings (Figure 1(a)) were dense and porous and NIP (Figure 1(c)) is smooth. The porous and consistent structure of coating indicates that the SPME-MIP fibers are suitable for effective analyte extraction from matrix samples. The FTIR spectra of the MIP and NIP fibers (Figure S1) show the C–O and C=O stretching vibration in the amide I or II of chitosan at 1045 and 1712 cm^−1^, respectively. The peak in 1238 cm^−1^ represented the C–O–C stretching. The two absorption peaks at 2868 cm^−1^ and 2939 cm^−1^, respectively, correspond to -CH_3_ and -CH_2_ asymmetric stretching of chitosan. The signal was identified at 3424 cm^−1^ in NIP spectra due to NH_2_ symmetry and O-H stretching.

TGA analysis represents the thermal stability of the polymer. The TGA curve (Figure S2) reveals a multistage decomposition with a minor mass drop at ∼100°C, because of the desorption of physically absorbed water. The weight loss at three different phases (between 150 and 700°C) is most likely due to the loss of functional groups. Also, because of the decomposition of the polymer network, the MIP shows a weight loss of around 450–600°C, such that the remaining weight at 800°C was around 76%.

Characterization of fibers by EDX (Figure S3) revealed the presence of carbon (C), nitrogen (N), and oxygen (O) with percentage compositions of 58.78, 15.80, and 20.51% (w/w), respectively.

### 3.2. Optimization of SPME Conditions

**Figure 2:**
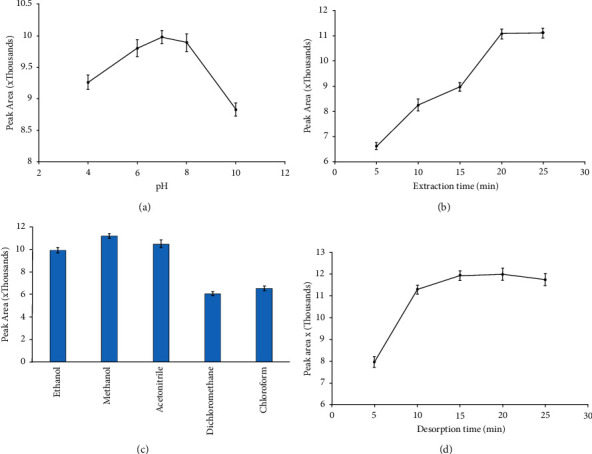
(a) Effect of pH on the extraction efficiency of SPME-MIP fiber for phenobarbital sample, extraction conditions: concentration of analytes; 0.1 *μ*g·mL^−1^, desorption solvent MeOH; extraction time: 20 minutes; desorption time: 15 minutes; stirring rate: 600 rpm, (b) effect of extraction time, extraction conditions: pH 7 and others are the same as (a), (c) effect of desorption solvent, extraction conditions: extraction time 20 minutes and others the same as (b) and (d) effect of desorption time, extraction conditions: the same as (c) and extraction solvent: MeOH.

The extraction efficiency of SPME-MIP fibers was enhanced by the optimization of several influential parameters such as sample pH, desorption solvent, extraction time, and desorption time. The effect of pH on adsorption properties was studied using phosphate buffer (0.1 M) with pH values ranging from 4 to 10 [32]. As demonstrated in Figure 2(a), extraction efficiency increased with increasing pH until pH 7 and then decreased as pH was raised further. In general, changing the pH of the sample matrix changes the electric charge of the species. Therefore, acidic and basic environments (pH less or more than pK_a_ of phenobarbital (7.63) inhibit analyte adsorption to the polymer, because the phenobarbital and amine groups of the chitosan convert to positive protonated or negative forms, respectively. Phenobarbital and polymer, on the other hand, are almost certainly in molecular form at pH values near to the pKa. This environment increases the likelihood of strong hydrogen bonding between the phenobarbital and the amine and hydroxyl functional groups, resulting in elevated extraction efficiency. As a result, in the following experiments, pH 7 was used as the best value.

Another major interfering component is the extraction time. When a fiber is exposed to a sample, it takes a certain length of time for the sample components to be extracted from the fiber coating. It has been established that in the equilibrium, the amount of analyte on the fiber reaches its maximum level [33]. The extraction time was varied from 5 to 25 min, and the results indicated that the extraction equilibrium was reached at 20 min (Figure 2(b)). As a result, future extraction trials were limited to 20 minutes in order to achieve maximum efficiency and minimize analytical time. After extraction, the fiber was recovered and rinsed with DDW to remove nonspecific binds, and then, desorption was carried out with the suitable solvent.

An important stage in the direct SPME approach is selecting a suitable eluent, especially when an off-line HPLC method is used. The influence of desorption solvent on SPME-MIP fiber with different solvents such as methanol, ethanol, acetonitrile, chloroform, and dichloromethane was also investigated. Methanol shows the highest recovery due to the polarity of phenobarbital (Figure 2(c)). Desorption time was also measured at 5-, 10-, 15-, 20-, and 25-minute intervals. Because the amount of desorbed phenobarbital did not differ significantly between 15 and 20 minutes in the subsequent studies, 15 minutes was chosen (Figure 2(d)). However, because agitation of the sample solution reduces extraction time, we chose 600 rpm as the optimal speed based on tests conducted on stirring rates ranging from 400 to 700 rpm.

### 3.3. Selectivity of the Prepared Fibers

**Figure 3:**
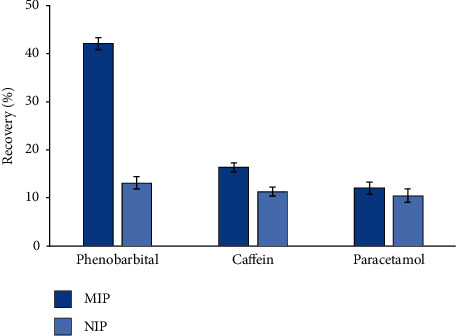
Comparison of the extraction efficiency of the phenobarbital SPME-MIP and NIP fiber in the presence of caffein and paracetamol, extraction conditions: pH 4.5; desorption solvent: MeOH: AcOH (9 : 1, v/v); extraction time: 30 min; desorption time: 20 minutes; stirring rate: 500 rpm.

The selectivity of the fibers was tested using phenobarbital and drugs with similar chemical structures, such as caffeine and paracetamol. The sample solution was spiked with 0.1 *μ*g mL^−1^ phenobarbital (and the other compounds), and then, extraction was performed with the SPME-MIP fiber. The HPLC conditions were the same as those in section 2.2. The extraction efficiency for phenobarbital using the SPME-MIP fiber was significantly higher than the other two identical compounds, as well as the SPME-NIP fiber's result (Figure 3). Also, it was observed that chemical adsorption on SPME-NIP fibers is nonselective, as evidenced by the equal extraction of caffeine and paracetamol by imprinted and nonimprinted fibers. It also underlines that imprinted fibers have a stronger selectivity because of specific cavities.

### 3.4. Extraction Capacity

**Figure 4:**
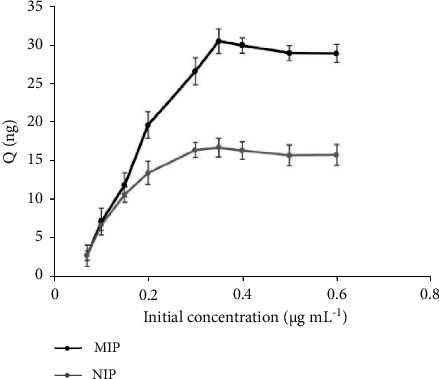
Extraction amounts curves for SPME-MIP and NIP fibers versus phenobarbital concentration. Extraction conditions: pH 7; desorption solvent: MeOH; extraction time: 20 minutes; desorption time: 15 minutes; stirring rate: 600 rpm.

To investigate the binding capacity of phenobarbital by the proposed method, the SPME-MIP and NIP fibers were immersed into a 7.0 mL of aqueous sample solution (pH adjusted to 7) at a concentrations range of 50–600 *μ*g·L^−1^. The amount of phenobarbital adsorbed by the SPME-MIP and NIP fibers was calculated using the following equation:

(1) $$\begin{array}{c} Q_{MIP}=\frac{\left(C_{i}-C_{f}\right)V}{n}, \end{array}$$

where *Q* (*ng*) is the sorption amount of phenobarbital, *C*_i_ (ng mL^−1^) is the initial concentration of phenobarbital, *C*_f_ (ng·mL^−1^) is the supernatant concentration of phenobarbital after adsorption, and V (mL) is the total volume of sample solution and n is the number of MIP fibers used. The isotherms of SPME-MIP and NIP fiber adsorption equilibrium for phenobarbital are shown in Figure 4. According to the calculations, the maximum adsorption quantities for the SPME-MIP and NIP fibers were 30.49 ng and 16.67 ng, respectively. On the other hand, the adsorption capacity of SPME-MIP fiber was calculated to be 112.95 *μ*g/g.

### 3.5. Adsorption Tests

The binding isotherm of analyte on SPME-MIP fibers is generally explained by Freundlich and Langmuir models, which is given in the following equation:

(2) $$\begin{array}{c} \frac{C_{e}}{q_{e}}=\frac{1}{q_{\max}K_{L}}+\frac{C_{e}}{q_{\max}},\\ \text{Log}q_{e}=\log\;K_{F}+\frac{1}{n\text{Log}C_{e}}, \end{array}$$

where *q*_e_ (ng) and *q*_max_ (ng) are the equilibrium and the maximum adsorption amounts of adsorbents, respectively. The Langmuir constant, which is related to binding site affinity and adsorption energy, is abbreviated as *K*_L_. The slope and intercept of the linear plot of *C*_e_/*q*_e_ versus C_e_ are used to compute the amount of *q*_e_ and *K*_L_. The linear plot between Log *q*_e_ and Log *C*_e_ yields the *K*_F_ constant (Freundlich constant) and *n*, which indicate the adsorption capacity and intensity, respectively. This research contributes to the understanding of the analyte's adsorption behavior on the fiber surface, as well as the evaluation of imprinting memory. For this purpose, static adsorption experiments were carried out in different concentrations of phenobarbital (0.2 to 0.6 *μ*g·mL^−1^). The Langmuir equation, with linear correlation coefficients of 0.944, was shown to be the best model for assessing adsorption behavior (Table S1). The findings confirmed the SPME-adsorptive MIP's sites' heterogeneity.

### 3.6. Analytical Performance of the Method

Several experiments were carried out to evaluate the proposed SPME-MIP method in terms of linearity, the limit of detection (LODs), single fiber repeatability, and fiber-to-fiber reproducibility. The calibration curve was linear in the range of 0.01–4 *μ*g·mL^−1^ with the correlation coefficient of 0.9979 (Figure S4). The *S*_b_ and *m* are the standard deviation of the blank and the slope of the calibration graph with values 9.09 and 3635.1, respectively. Therefore, the value of LOD (3*S*_b_/m) was 7.50 ng·mL^−1^ for phenobarbital and limit of quantification (LOQ, 10*S*_b_/m), which was the lowest concentration that could be quantitatively detected, was 25.0 ng·mL^−1^ (Table 1, Figure S5). The fiber-to-fiber repeatability was 5.90%, for three different fibers produced under identical conditions. After analyte desorption, a blank analysis was performed to assess any possible carry over effect from prior extractions. The results showed that phenobarbital was entirely desorbed from the sorbent and no carryover effect was seen in the blank analysis. The enrichment factors, which is defined as the ratio of phenobarbital concentrations after extraction to concentrations before extraction, was 46.4 and the extraction efficiency obtained 66.2% under optimum conditions. These data, together with prior findings, support the method's relatively excellent analytical performance.

### 3.7. Application of SPME-MIP in Environmental Samples

**Figure 5:**
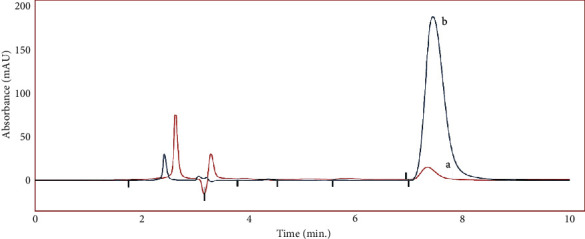
Typical chromatograms of (a) direct injection of spiked well water sample and (b) spiked well water sample after SPME-MIP procedure. Spiked concentration: 0.1 *μ*g·mL^−1^.

To validate the manufactured SPME-MIP fiber, several water samples were collected from diverse sources (river and well). 7 mL water samples were extracted using the SPME-MIP fiber in optimum conditions. A 0.45-*μ*m membrane was used to filter the samples prior to analysis. Phenobarbital was not found in any of the samples in the initial tests (Table 2). The water samples were then spiked with 0.015, 0.1, 0.5, and 1 *μ*g/mL of phenobarbital.  Figure 5 shows the chromatogram of a well water sample after being spiked with a standard solution. The clear chromatogram confirms the specific extraction of phenobarbital.

In addition, the determination of phenobarbital at various concentration levels was used to test the repeatability and reproducibility (intraday and interday precisions). The recovery of phenobarbital at various spiking doses ranged from 92.4 to 98.0 percent (calculated by comparing the determined and added amounts to the real samples). In addition, 2.40–4.28% intraday and 3.15–6.45% interday precisions were found. These data show that the produced SPME-MIP fiber can efficiently and quickly separate phenobarbital from water samples.

### 3.8. Comparison with Other Methods for Phenobarbital Determination

The analytical capabilities of the SPME-MIP fiber were compared to a number of different methods for analyzing various sample matrices. Our proposed approach has a low LOD and a high recovery compared to the majority of the methods indicated in the table (Table 3). The proposed fiber shows high efficiency (due to the high selectivity of SPME-MIP fibers), as well as the convenience and low cost of fiber synthesis. On the other hand, the maximum adsorption amount using the SPME-MIP fiber used in this work for phenobarbital determination was 112.95 *μ*g·g^−1^ , which was lower than the reported amounts in literature studies, such as Haginaka et al. (812.82 *μ*g·g^−1^) using the magnetic molecularly imprinted polymer (M-MIP) [27].

## 4. Conclusions

In this study, using chitosan as a natural monomer and glutaraldehyde (GLA) as a chemical cross-linking agent, the SPME-MIP fiber was produced on the surface of modified SS wire and used to detect phenobarbital. This method offers a number of advantages, including its simplicity and speed. By using natural and environmentally friendly biopolymer, polymerization was carried out under mild conditions and reduced use of chemicals. Furthermore, because of using the stainless-steel core, the manufactured SPME-MIP fiber displays higher mechanical characteristics than conventional SPME fibers. The developed polymer, when paired with the SPME method, improves the sample preparation method due to chitosan's significant features. The SPME-MIP fiber was used to successfully extract phenobarbital from natural water samples. However, because this approach has a poor sensitivity, future research could focus on developing new sorbents on stainless-steel wires to improve the sensitivity of the SPME fiber.

## Figures and Tables

**Table 1:** Analytical characteristics of the proposed method

Figures of merit	
R^2 a^	0.9979
Linear range (*μ*g·mL^−1^)	0.01-4
LOD (ng·mL^−1^)^b^	7.50
LOQ (ng·mL^−1^)	25.0
A single fiber (RSD)% ^c^	3.89
Fiber to fiber (RSD)%	5.90

^a^Square of correlation coefficient. ^b^Limit of detection (*n* = 20). ^c^Relative standard deviation for 6 replicate determinations of 0.1 *μ*g·mL^−1^ phenobarbital.

**Table 2:** Analysis of phenobarbital in spiked real water samples by SPME-MIP with HPLC-UV

Sample	Added (*μ*g·mL^−1^)	Founded (*μ*g·mL^−1^)	Recovery (%)	RSD (%)	
				Interday	Interday
River water	0	—	—	—	—
	0.015	0.0142	94.8	2.40	4.85
	0.1	0.0966	96.6	2.73	3.15
	0.5	0.4700	94.0	3.59	4.58
	1	0.9750	97.5	4.28	5.38
Well water	0	—	—	—	—
	0.015	0.0138	92.4	4.04	4.58
	0.1	0.0954	95.4	3.76	5.28
	0.5	0.4775	95.5	3.87	6.45
	1	0.9800	98.0	4.16	5.07

**Table 3:** Comparison with reported methods for the determination of phenobarbital

Extraction method	Detection	Linear range (*μ*g·mL^−1^)	LOD (ng·mL^−1^)	Recovery (%)	Q^h^ (*μ*g·g^−1^)	RSD (%)	References
SPME^a^	HPLC-UV	0.0005–5	0.32	102	—	2.9	[5]
MIS-SPE^b^	HPLC-UV	10–100	10000	41–75	—	—	[28]
SFODME^c^	HPLC-UV	0.002–0.3	1	95.8–98.8	—	4.4	[3]
MIPs-GSCDs^d^	Spectrofluorometer	9.2 × 10^−5^–0.008	0.023	-	—	7.3	[29]
SPME^a^	GC-MS	0.25–25	150	94.6–106.0	—	4.2–7.7	[34]
SPME^a^	GC-MS	0.2–40	200	—	—	6.3–7.7	[24]
MMIP^e^	HPLC-UV	1–80	—	—	812.82	—	[27]
SBSE^f^	HPLC-UV	0.08–40	80	72–78	—	5.2–7.6	[35]
SPME-MIP^g^	HPLC-UV	0.01–4	7.50	92.5–98.0	112.95	2.4–4.2	This work

^a^Solid-phase microextraction. ^b^Molecularly imprinted silica—solid-phase extraction. ^c^Solidified floating organic drop microextraction. ^d^Molecularly imprinted polymers—green source carbon dots. ^e^Magnetic molecularly imprinted polymer. ^f^Stir bar sorptive extraction. ^g^Solid-phase microextraction molecularly imprinted polymer. ^h^Adsorption capacity.

## Data Availability

The data used to support the findings of this study are included within the article and the supplementary information file.

## Supplementary Materials

Supplementary Materials Figure S1. The FTIR spectra of (a) MIP and (b) NIP. Figure S2. TGA profile curve under N_2_ atmosphere at 10°C·min^−1^. Figure S3. EDX spectra of phenobarbital-MIP-SPME fiber. Figure S4. HPLC chromatograms of phenobarbital with SPME-MIP (0.01–4 *μ*g·mL ^−1^). Figure S5. HPLC chromatograms of phenobarbital with SPME-MIP (0.025 *μ*g·mL ^−1^). Table S1. Parameters of Langmuir and Freundlich equations.
