# Supplementary material for: Preparation of Molecular Imprinted Polymer Based on Chitosan as the Selective Sorbent for Solid-Phase Microextraction of Phenobarbital
Source: J Anal Methods Chem. 2022 Jul 13;2022:9027920. doi: 10.1155/2022/9027920 (PMC9300383; doi:10.1155/2022/9027920)
Supplement: Supplementary Materials — Figure S1. The FTIR spectra of (a) MIP and (b) NIP. Figure S2. TGA profile curve under N2 atmosphere at 10°C·min−1. Figure S3. EDX spectra of phenobarbital-MIP-SPME fiber. Figure S4. HPLC chromatograms of phenobarbital with SPME-MIP (0.01–4 μg·mL −1). Figure S5. HPLC chromatograms of phenobarbital with SPME-MIP (0.025 μg·mL −1). Table S1. Parameters of Langmuir and Freundlich equations. [file 9027920.f1.doc]

Electronic Supporting Material

**Preparation of molecular imprinted polymer based on chitosan as the selective sorbent for solid phase microextraction of phenobarbital**

**Marzieh Rahimi^a^, Soleiman Bahar^a,*^, S. Mojtaba Amininasab^a^**

*^a^Department of Chemistry, Faculty of Science, University of Kurdistan, P.O. Box 416 Sanandaj, I.R. IRAN*

*^*^ Corresponding author**at: University of Kurdistan, Department of Chemistry, Sanandaj, Iran.*

*Tel.: +98 8733664600; Fax: +98 8733664600; E-mail address: : s.bahar@uok.ac.ir*

*^a^Department of Chemistry, Faculty of Science, University of Kurdistan, P.O. Box 416 Sanandaj, I.R. IRAN*

*^*^ Corresponding author**at: University of Kurdistan, Department of Chemistry, Sanandaj, Iran.*

*Tel.: +98 8733664600; Fax: +98 8733664600; E-mail address: : s.bahar@uok.ac.ir*


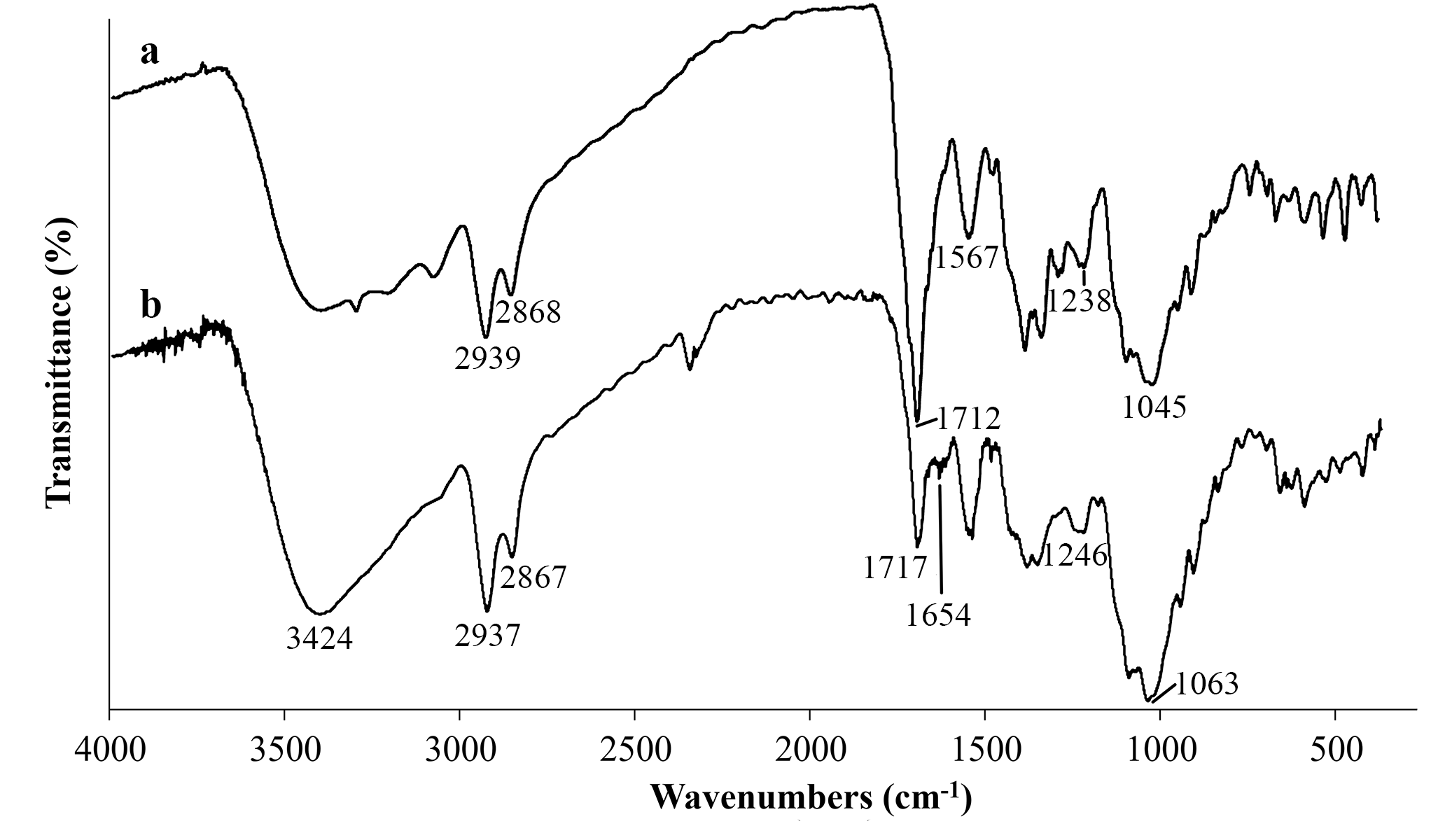


**Fig. S1** The FTIR spectra of a) MIP and b) NIP.


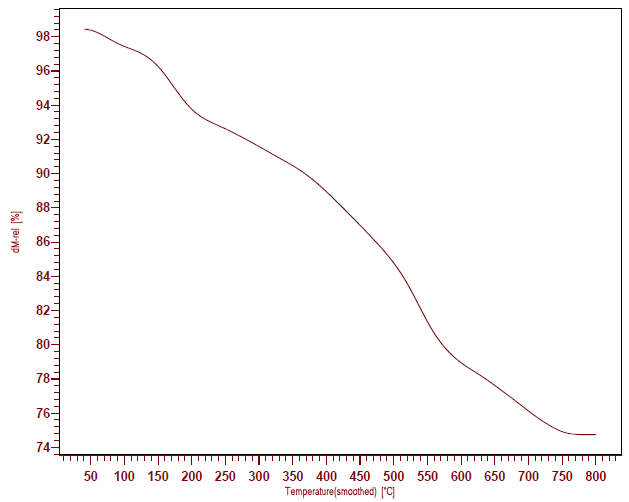


**Fig. S2** TGA profile curve under N_2_ atmosphere at 10 ˚C min^-1^.

**Fig. S3** EDX spectra of phenobarbital-MIP-SPME fiber.


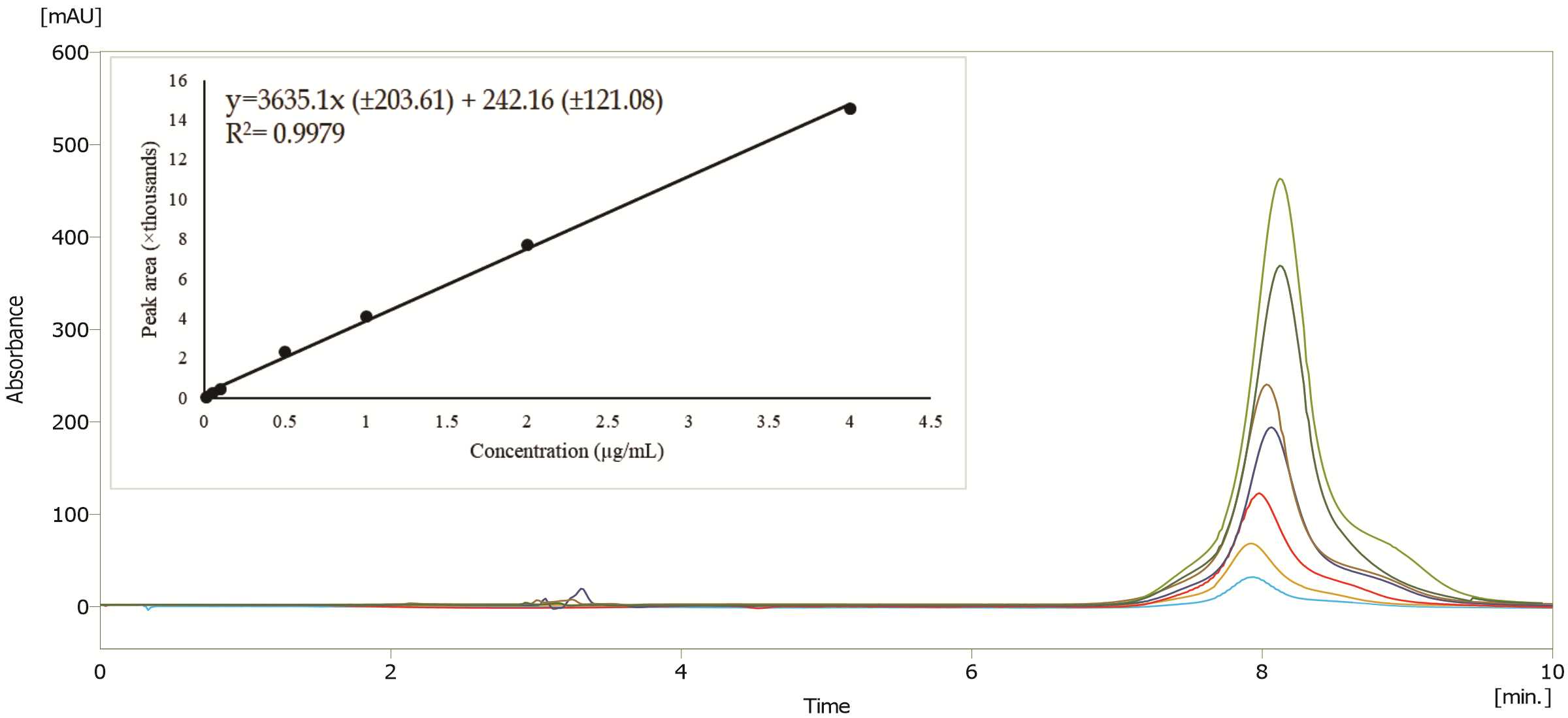


**Fig. S4** HPLC chromatograms of phenobarbital with SPME-MIP (0.01- 4 µg mL ^-1^).


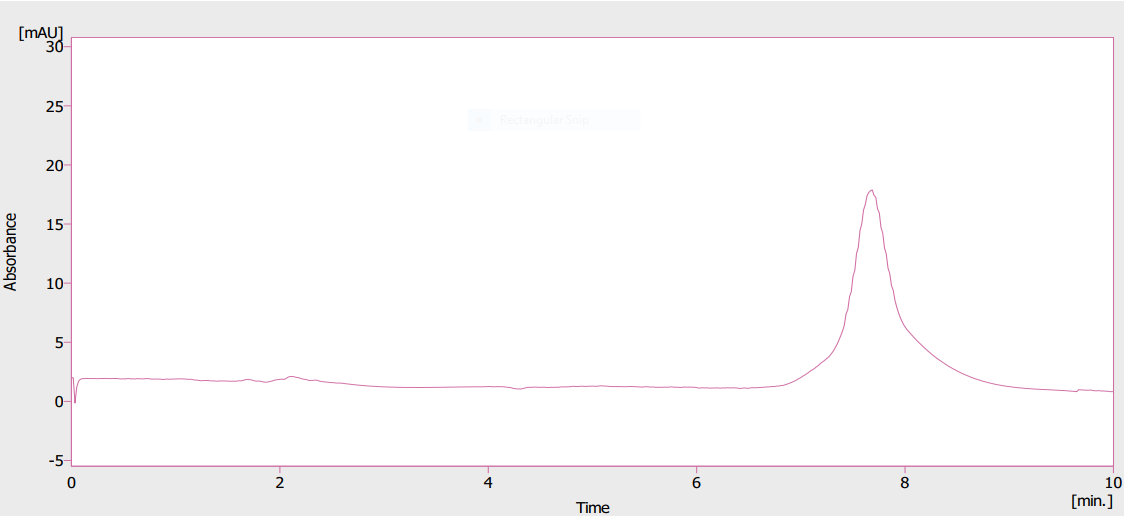


**Fig. S5** HPLC chromatograms of phenobarbital with SPME-MIP (0.025 µg mL ^-1^).

**Table S1** Parameters of Langmuir and Freundlich equations

| Langmuir | | | Freundlich | | |
| --- | --- | --- | --- | --- | --- |
| K_L_ | q_max_ | R^2^ | K_F_ | n | R^2^ |
| 0.00001 | 34.7 | 0.944 | 1.15 | 3.24 | 0.604 |
